# The State of the Art and Practice in Digital Preservation

**DOI:** 10.6028/jres.107.010

**Published:** 2002-02-01

**Authors:** Kyong-Ho Lee, Oliver Slattery, Richang Lu, Xiao Tang, Victor McCrary

**Affiliations:** National Institute of Standards and Technology, Gaithersburg, MD 20899-8951

**Keywords:** digital preservation, emulation, encapsulation, migration, standardization, XML

## Abstract

The goal of digital preservation is to ensure long-term access to digitally stored information. In this paper, we present a survey of techniques used in digital preservation. We also introduce representative digital preservation projects and case studies that provide insight into the advantages and disadvantages of different preservation strategies. Finally, the pros and cons of current strategies, critical issues for digital preservation, and future directions are discussed.

## 1. Introduction

Information preservation is one of the most important issues in human history, culture, and economics, as well as the development of our civilization. While earliest information was recorded in carvings on stone, ceramic, bamboo, or wood, the development of civilization paved the way for new storage media and techniques for recording information, such as writing on silk or printing on paper. Eventually we were able to put photographic images on film and music on records. A revolutionary change occurred in the information storage field with the invention of electronic storage media.

With the advent of high-performance computing and high-speed networks, the use of digital technologies is increasing rapidly. Digital technologies enable information to be created, manipulated, disseminated, located, and stored with increasing ease. Ensuring long-term access to the digitally stored information poses a significant challenge, and is increasingly recognized as an important part of digital data management [1,2].

**Fig. 1:**
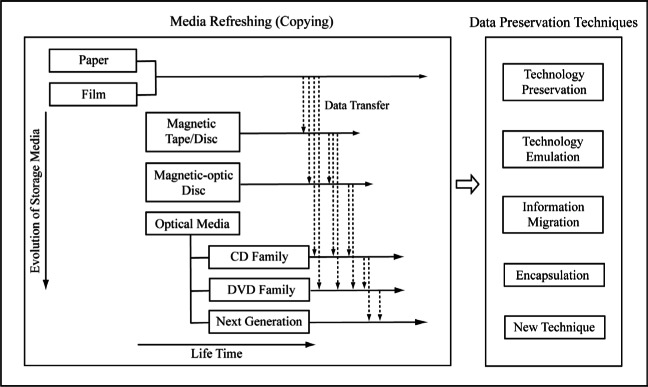
A progression of information preservation strategies.

The evolution of data storage media and the development of the preservation technology can be described as shown in Fig. 1. This diagram lists the various media used in data storage (both digital and analog) and the techniques needed to ensure that the data on them is preserved. It also highlights the trend from analog to digital/optical storage media and indicates the transfer of data from one generation of media to the next. It is clear that while it is easier to create, amend, and distribute digital data, the media storing this data such as optical discs are not as robust as traditional analog media such as paper or film. In view of modern information preservation requirements, this paper will focus on the aspects of the technical strategies used in digital information preservation.

Digital preservation involves the retention of both the information object and its meaning. It is therefore necessary that preservation techniques be able to understand and re-create the original form or function of the object to ensure its authenticity and accessibility. Preservation of digital information is complex because of the dependency digital information has on its technical environment. Furthermore, as newer digital technologies rapidly appear and older ones are discontinued, information that relies on obsolete technologies soon becomes inaccessible. Therefore, digital resources present more difficult problems than conventional analog media such as paper-based books [3].

Recently, several approaches for digital preservation have been identified and presented. Conventional methods are mainly technology emulation, information migration, and encapsulation [4,5,6,7,8]. However, there is a lack of proven preservation methods to ensure that the information will continue to be readable.

To promote a solid understanding of the pros and cons of different preservation techniques, this paper tries to present a comprehensive survey of them through a review of a wide range of literature and representative projects. We suggest a preservation strategy based on XML (eXtensible Markup Language) [9] for interoperability and interchangeability of preserved digital information. The most appropriate preservation strategy should be determined by considering various aspects including cost-effectiveness, legal restrictions, and user access requirements. However, as stated earlier, this paper particularly focuses on the technical issues of preservation.

This paper is organized as follows. Section 2 summarizes various techniques for digital preservation. Their advantages and disadvantages are presented through a literature survey. By introducing current projects and case studies, the application of these preservation techniques is described in detail in Sec. 3. Finally, points of discussion and a summary are given in Sec. 4.

## 2. Digital Preservation Techniques

This section introduces the current strategies for digital preservation. Techniques for the preservation of digital information include technology preservation, technology emulation, information migration, and encapsulation.

Digital resources can be stored on any medium that can represent their binary digits or bits, such as a CD-ROM or a DVD. Rothenberg [10] defines a bit stream as an intended meaningful sequence of bits with no intervening spaces, punctuation, or formatting. To preserve that bit stream, the first requirement is to ensure that the bit stream is stored on a stable medium. If the digital medium deteriorates or becomes obsolete before the digital information has been copied onto another medium, the data will be lost.

Therefore, digital preservation involves copying the digital information onto newer media before the old media becomes so obsolete that the data cannot be accessed. This is referred to as copying or refreshing [6,8]. This process preserves the integrity of the digital information. Well-established techniques for preserving the integrity of the digital information exist at this level [5].

For timely refreshing, the lifetime of digital storage media must be predicted. The lifetime of a medium determines the period of time in which the information recorded on the medium is stored safely without loss. The specification of the lifetime of the medium will prompt librarians or archivists to refresh their media before medium deterioration. Recently, Zwaneveld [11] suggested that media could be rated into five classes according to certain criteria including the lifetime.

However, simply copying the digital information is not always sufficient as a preservation strategy [5]. One has to ensure that the information can be retrieved and processed in the future. Retrieving a bit stream requires a hardware device such as a disk drive for reading the physical representation of the bits from the medium, as well as driver software.

**Fig. 2:**
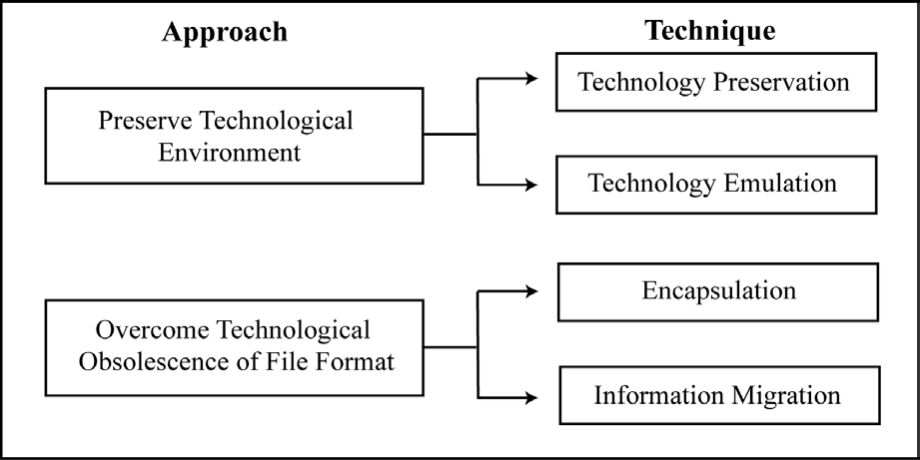
Existing preservation approaches.

Existing preservation strategies can be broadly classified into two main approaches as shown in Fig. 2. The first is the more conservative approach where the original technological environment is fully preserved for decoding the digital information in the future. This approach can be further divided into two preservation techniques. The first is to preserve the working replicas of all computer hardware and software platforms for future use. This is referred to as the technology preservation strategy [5]. The other is to program the newer computer systems to emulate on demand the older obsolete platforms and operating systems. This is the so-called technology emulation strategy [10].

The second approach is to overcome the technical obsolescence of file formats. It may also be classified into two techniques. The first is to transform or convert the old digital resource to a format that is independent of the particular hardware and software that were applied to create them. This is called the information migration strategy [5]. The second technique, termed encapsulation, is where a digital object and anything else necessary to provide access to that object are grouped together and preserved [12].

### 2.1 Technology Preservation

This solution supposes that complete museums of obsolete equipment could be maintained in order to replicate any old configuration of hardware and software [13]. This strategy involves preserving an original application program, operating system software, and hardware platform [6].

The advocates of this strategy emphasize that the original environment needs to be run to really preserve the behavior as well as the look and feel of the digital object. For some digital objects, this may be the best solution at least in the short-term because it ensures that the material is accessible by preserving the access tools as well as the object itself.

However, various issues including space, maintenance, and costs may make this impossible in the longer term. Specifically, equipment ages and breaks, documentation disappears, vendor support vanishes, and the storage medium as well as the equipment deteriorates [14]. This strategy also limits the portability of the resource since it will depend on hardware stored in specific locations [3].

### 2.2 Technology Emulation

This strategy has a lot in common with the technology preservation strategy. It involves preserving the original application program. Emulator programs can be designed and run on future computer platforms. The emulator is programmed to mimic the behavior of old hardware platforms and operating system software, such as for games and executable files [6,16]. However, this strategy does not involve preserving ageing hardware and original operating system software.

**Fig. 3:**
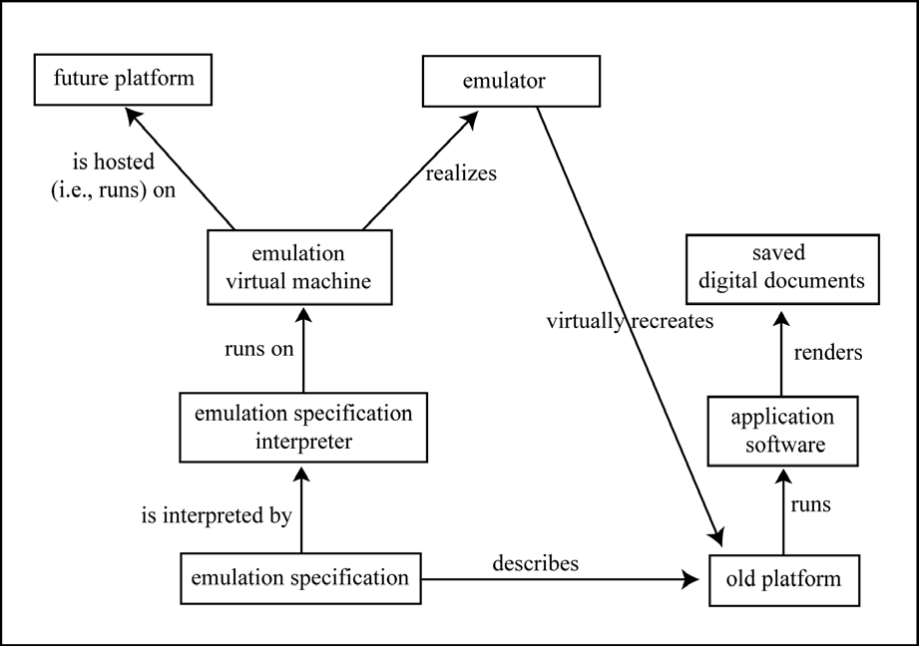
Rothenberg’s emulation-based preservation [15].

The goal of emulation is to preserve the look and feel of the digital object as well as its functionality. The essence of this strategy is to copy the technical context of the resource allowing the original object or a refreshed copy of the original object to be used in the future. Rothenberg illustrates the conceptual view of the relationships among elements of the emulation process as shown in Fig. 3 [15]. This approach decouples application programs from the platform via a virtual machine with the result that applications can run on different platforms by migrating the virtual machine to those platforms. The Java virtual machine might be an example.

Hendley sees emulation as a short to medium term strategy or a specialist strategy where the need to maintain the physical presence of the original digital resource is of great importance to the users. Again, he sees technology emulation being primarily used in cases where digital resources cannot be converted into software independent formats and migrated forward.

Waugh et al. [12] indicate that the application software itself could contain viruses that would result in the loss of information over time. Particularly, they note that emulation requires preserving a significant amount of information. They consider it useful if the goal is to preserve the software as an artifact itself and if future user organizations lack sufficient knowledge to understand the format of the digital information. Granger [16] discusses its potential advantages such as the re-creation of the look and feel of the resource. They identify the possible disadvantages as the undefined nature of technological change and the complexity of creating emulator specifications. His conclusion is that emulation is not a complete digital preservation solution but a partial one.

On the contrary, Russell [3] states that emulation potentially offers the best solution for very long-term preservation of digital material, especially for resources for which the value is unknown and where future use of the material is unlikely. Woodyard [1] points out that emulation could be a more suitable solution than migration for long-term access to complex digital resources such as executable files. Gilheany [18] discusses the need for emulators to permanently preserve the functionality of computers. Rothenberg advocates and recommends emulation as the best possible solution [10,18] and presents results from the first phase of an emulation experiment [15] in the Networked European Deposit Library (NEDLIB)^1^ project [19].

Holdsworth and Wheatley [20] consider the method of Rothenberg, in which the production of an emulator can be postponed by instead producing an emulator specification at the time of platform obsolescence, as a very risky approach to preserving information forever. This is because there is no guarantee that a specification produced can be used to produce an emulator in the future. They present some illustrative guidelines for the use of emulation and argue that emulation is a valid method of digital preservation, at least for systems where the documentation is not available in electronic form. Holdsworth [21] also suggests the use of widely available programming languages such as C in the implementation of emulation.

The emulation approach requires detailed specifications for the outdated hardware and operating system software [10]. Therefore, standard formats for the emulation specification need to be developed. The extent to which emulation should mimic the original technical environment entirely or emulates only those components necessary to access the data remains an issue for debate.

### 2.3 Information Migration

The migration of digital information refers to the periodic transfer of digital materials from one hardware and software configuration to another, or from one generation of computer technology to a subsequent generation [5]. The purpose of migration is to preserve the integrity of digital objects and to retain the ability of users to retrieve, display, and use them in the face of constantly changing technology.

The Open Archiving Information System (OAIS) model [22], developed by the Consultative Committee for Space Data Systems (CCSDS)^2^, breaks migration into four categories: refreshment, replication, repackaging, and transformation. Refreshment ensures that a reliable copy of the bit stream of a digital object is maintained while replication and repackaging ensure that a manageable package of the object is available. On the other hand, transformation actually modifies the bit stream of a digital object and this is what is considered as the process of migration in this paper.

Rather than focus on the technology, information migration tends to focus on the intellectual content and on ensuring its accessibility using current technology [3]. This strategy could be facilitated through copying the digital information to an analog medium or through application programs that are backward compatible. It could also be facilitated through the conversion of digital resources into a small number of standard formats that are hardware and software independent [6].

The first solution involves the transfer of digital resources from less stable to more stable analog media such as paper or microfilm [23]. While analog media have better proven long-term reliability, they may not provide adequate representations of the original object. This solution may also lead to severe loss in functionality and presentation of the original digital object. It is not possible, for example, to microfilm the equations embedded in a spreadsheet, to print out an interactive video, or to preserve a multimedia document as a flat file [5,18,24].

The second solution, software that is backward compatible, will simplify migration. The latest versions of most popular word processing packages, for example, will be able to decode files created on earlier versions of the same package. While this strategy may work over the short term for simple digital resources created on some of the leading application packages, it cannot be relied upon over the medium to long-term or for more complex digital resources. Meanwhile, interoperability of systems will also facilitate migration because a specific program is not needed to access a digital resource. However, these features become harder to achieve with greater software complexity. Hence, any information migration through backward compatibility or interoperability between application software packages would represent a short-term strategy [6].

Another approach entails initially migrating digital information from the great multiplicity of formats to a smaller more manageable number of standard formats. These are less volatile than the wide array of nonstandard formats and can still encode the complexity of structure and form of the original resource [5]. Decisions on which formats to convert digital resources into should be based on the structure of the digital resources, on the objectives set by the collection manager, and on the requirements of the users of that collection [6]. For example, one important issue is whether the top priority is given to preserving the ability to process the digital resource or to preserving the format or visual presentation of the digital resource.

A report [5] from the task force on the Commission on Preservation and Access (CPA) and the Research Libraries Group (RLG) makes the point that there are a variety of migration strategies. Particularly, Wheatley [25] attempted to break migration into more specific cases in the Creative Archiving at Michigan and Leeds: Emulating the Old and the New (CAMiLEON)^3^ project: minimum migration, preservation migration, recreation, human conversion migration, and automatic conversion migration. He applied each case to test materials and discussed its usefulness for digital preservation.

Recently, the concept of migration on request has been conceived by the Consortium of University Research Libraries Exemplars for Digital Archives (CEDARS)^4^ project [3], in which original objects are maintained and preserved in addition to a migration tool that runs on a current computing platform and can be employed by users as necessary. When the current platform becomes obsolete, the migration tool will no longer work. Therefore, the preservation problem in this case is obviously focused on the maintenance of the migration tool. Preserving the original bit stream and a migration tool is compatible with emulation strategy.

The task force report and Hendley consider this migration strategy the most promising for the future. Bearman [26] also believes that it is the most promising strategy for preservation of electronic records and the only one that has worked to date. Russell [3] points out that the migration strategy is the most practical approach at least for the short and medium term.

However, for resources where it is difficult to disentangle format from content such as complex multimedia documents, this is not an easy option. Multiple components may require separate migration activities and this can be very complex. Indeed, for some multimedia resources, migration may not be possible without significant compromises in functionality. Russell states that the costs of migration may, in the long run, exceed those costs necessary for preserving either the technology itself or the detailed technical specification that will allow future emulation.

Waugh et al. believe that the key to successful migration is the knowledge of the original data format and a close match in functionality between the original and replacement format. They consider migration the simplest over the short and medium term for digital information that is actively managed. On the other hand, Rothenberg dismisses the strategy of migrating electronic records systematically before they become inaccessible [18].

Lawrence et al. [27] have attempted to quantify the risks involved in the use of migration and have analyzed several commercially available migration tools for their relative accuracy. This practical investigation has led to the identification of some key requirements for migration software: access to the source file specification, analysis of the differences between it and the target format, identification of the degree of risk in the case of a mismatch, accurate conversion of the source file to a target specification, and so on.

However, none of current migration methods or tools comes close to meeting all of these requirements. It is important to realize that technical standards may change rapidly and that this strategy may not ensure that digital information remains accessible. Recently, however, XML has become widely accepted as a universal standard format in various fields of digital library, electronic commerce, and the Web. Standardization based on XML could be helpful in addressing the digital preservation problem.

### 2.4 Encapsulation

Encapsulation aims to overcome the problems of the technological obsolescence of file formats by making the details of how to interpret the digital object part of the encapsulated information. This strategy involves creating the original application that was used to create or access the digital object on future computer platforms. Part of the process of encapsulation may be to migrate the record to a more easily documented format.

The concept of encapsulation is similar to the Bento^5^ [28] container, which was developed to increase compatibility of data between computer applications. Bento is a specification for storage and interchange of compound content, and is designed to be platform and content neutral. Thus, it provides a convenient container for transporting any type of compound content between multiple platforms.

Encapsulation can be achieved by using physical or logical structures called containers or wrappers to provide a relationship between all information components such as the digital object and some supporting information including metadata [29,30]. The reference model for the OAIS also describes the types of supporting information that should be included in an encapsulation. They include the representation information used to interpret the bits appropriately, the provenance to describe the source of the object, the context to describe how the object relates to other information outside the container, a reference to one or more identifiers to uniquely identify the object, and fixity to provide evidence that the object has not been altered.

The Universal Preservation Format (UPF) [31,32,33] is a method being developed for digital preservation, based on the theory of encapsulation. It is a self-describing storage technology, which uses a wrapper to hold the digital object and the metadata together to protect against technological obsolescence. The Digital Rosetta Stone [34] is a method for storing the representation information needed to interpret the digital content of an object separate from the encapsulation to avoid duplication of effort and inefficient use of storage space.

Encapsulation has been widely promoted by Rothenberg who is a strong advocate of encapsulation and emulation methods for digital preservation. Day [30] and Shepard [35] have also supported the encapsulation approach. However, Bearman [26] disputes the theory of Rothenberg stating that it is not clear as to how metadata encapsulation strategies may be practically implemented.

Waugh et al. argue that encapsulation is the best basis for long-term preservation. They introduce three challenges of encapsulation: the requirements for applications to generate encapsulated records, the potential storage overhead of including documentation about the format within each record, and information about unpublished data formats. However, even if format specifications are publicly available, they are often incomplete and substantial components of file specifications often consist of nonstandard elements.

Encapsulation can be considered to be a type of migration technique. Although documentation may delay the need for migration for a long time, the encapsulated information will eventually need to be migrated. Therefore, encapsulation techniques can be applied to the digital resources whose format is well known and that are unlikely to be accessed actively.

### 2.5 The Digital Tablet

Kranch [36,37] proposes developing a digital tablet for preservation. The digital tablet technique does not precisely fit into the above categories. The tablet would have a self-contained power source, present the stored digital information on a screen as glyphs from a written language appropriate for the information, and have touch-sensitive controls to change the presentation and manipulate the information. Additionally, it should be able to withstand millennia of neglect under harsh conditions yet cost no more than a few dollars to produce and encode. It should have a storage capacity of dozens or hundreds of terabytes, and contain a simple serial read-only port to download the original digital information into an external system along with instructions on how to do it. However, the digital tablet may be considered to be another technology preservation method.

## 3. Projects and Case Studies

This section introduces representative projects for digital preservation, describes their goals and results, and presents their specific preservation strategies. Recently, Cloonan and Sanett [38] presented a report on the initial phase of projects involved in developing, evaluating, and/or implementing digital preservation strategies in Europe, Australia, and the United States.

### 3.1 Australian Projects

Australia has been examining digital preservation issues since 1994. Several projects are aiming to preserve various digital materials such as electronic records, online publications, digital audio resources, theses, and cartographic materials [1,12,39]. The Victorian Electronic Records Strategy (VERS)^6^ project [12] produced a standard for management and preservation of electronic records [40,41]. The standard proposed by the VERS project recommends encapsulating the documents and their context in a single object based on XML.

The Preserving and Accessing Networked Documentary Resources of Australia (PANDORA)^7^ project by the National Library of Australia has led the way in archiving the Web [42,43]. The primary objective of PANDORA is to capture, archive, and provide long-term access to significant online publications. The project aims at addressing both archiving and preservation processes. The PANDORA archiving processes refer to the collection and provision of immediate access to the publications while preservation processes involve managing the materials and applying appropriate strategies (e.g., migration) to ensure long-term access. The archiving processes have been developed while techniques for managing long-term access to these digital resources is still being developed.

The project has also embarked on migration experiments with some HTML pages. The format of these pages is not yet obsolete, but the HTML specification [44] has declared a number of mark-up tags as dead and not to be supported in this or future versions. The aim of these trials is to make changes in the HTML source code to remove tags declared as dead and replace them with current tags, effectively migrating the source code to a different version to reduce problems of future compatibility with Web browsers. Additionally, the National Archives of Australia is undertaking a project to develop advice for commonwealth agencies on using migration as a preservation strategy for electronic records [45].

### 3.2 CEDARS

The Consortium of University Research Libraries, which represents both university and national libraries across the UK and Ireland, leads the CEDARS project. The project is based on the OAIS model described earlier. Essentially it is an archival model for an archiving system but does not explicitly include a preservation module. Current projects investigating the use of the model include NEDLIB in Europe, CEDARS in the UK, and PANDORA in Australia.

The CEDARS project aims to address the strategic, methodological, and practical issues of digital preservation as well as providing guidance for libraries in best practices. Specifically, the project is producing guidelines for developing digital collection management policies and preserving different classes of digital resources. Additionally, it is performing an analysis of the cost implications of digital preservation. The project is running pilot projects to test and promote the chosen strategy for digital preservation. The CEDARS Data Preservation Strategies Working Group is looking at preservation issues that are related with migration, emulation, and data refreshing.

Specifically, the project has done some work comparing migration and emulation across older digital materials in conjunction with the CAMiLEON project [20,25]. They suggest that both migration and emulation strategies are viable for different types of digital materials. They also believe that no strategy is a panacea, and the strategy adopted for providing access to preserved resources will very much depend on the nature of the resource itself and the reason for its preservation. More work is planned to investigate the information loss associated with each strategy.

### 3.3 CAMiLEON

The CAMiLEON project is funded by the Joint Information Systems Committee (JISC) in the UK and the National Science Foundation (NSF) in the USA. The project looks at the issues surrounding the implementation of technology emulation as a digital preservation strategy. The project recognizes the potential of emulation for the retention of the functionality and the look and feel of digital objects [20]. It aims to develop tools, guidelines, and costs for emulation as compared to other digital preservation options.

The project is performing user testing of various digital resources both in their original environments and in emulated environments. The project presents guidelines for use of emulation and argues that emulation is a valid method for both complex digital resources that include executable files and resources for which the documentation is not available in electronic form.

### 3.4 NEDLIB

The NEDLIB project was initiated by a permanent standing committee of the Conference of European National Libraries (CENL) in 1998, with funding from the European Commission’s Telematics Application Programme. Eight national libraries in Europe, one national archive, and major publishers are participating in the project. The National Library of the Netherlands leads the project.

The project aims to develop a common architectural framework and basic tools for building deposit systems for electronic publications. The project has also adopted the OAIS migration model described earlier. The main objective of the NEDLIB project is to provide better insight into the merit and weakness of different long-term preservation strategies. The project defines the characteristics of electronic publications and other categories of digital deposit material and associated preservation and authenticity requirements. It is recognized that many aspects including cost-effectiveness, legal restrictions, agreements with publishers, and user access requirements ultimately need to be taken into account when policy choices for preservation strategies are set. However, the project focuses on the technical issues of preservation.

The project has taken a first step to test the technicalities of the preservation mechanisms by starting an emulation experiment. The fundamental idea of the work is to test whether emulating obsolete computer hardware on future systems could be used to ensure long-term access to digital publications. Rothenberg has performed the first phase of this experimental work [15]. It involved developing a prototype experimental environment for trying out emulation-based preservation and using commercial emulation tools to provide an initial proof-of-concept. The experimental results indicate that emulation should work in principle, assuming that suitable emulators for obsolete computing platforms can be hosted on future platforms.

### 3.5 Kulturarw^3^ Heritage

The Kulturarw^3^ Heritage Project^8^ of the Royal Library in Sweden is testing methods of collecting, archiving, and providing access to Swedish electronic documents. Web crawlers or robots are used in order to collect all the Swedish Web pages automatically. Although the project currently does not focus on preservation, it is growing into a broader Nordic initiative that may explore the long-term preservation of this archive.

### 3.6 Library of Congress

Recently, the Computer Science and Telecommunications Board (CSTB) of the National Academies [46,47] convened the Committee on the Information Technology Strategy for the Library of Congress for advice on digital preservation. The committee report includes specific recommendations for enhancing technology infrastructure, particularly in the area of networks, databases, and information technology security.

The Library of Congress’ pilot project, working with the Internet Archive^9^, has worked through all aspects of archiving the Web in the area of political Web sites. This project uses the Digital Library SunSITE Collection and Preservation Policy^10^ from the University of California, Berkeley, which provides several digital collecting levels, as guidance.

### 3.7 NARA: Persistent Archives and Electronic Records Management

The NARA (National Archives and Records Administration) project,^11^ which is led by the San Diego Supercomputer Center and funded by NARA, aims to develop a persistent archive to support ingestion, archival storage, information discovery, and preservation of digital collections. One of its premises is the importance of preserving the organization of digital collections simultaneously with the digital objects that comprise the collection [48].

The ultimate goal is to preserve not only the original data, but also the context that permits the data to be interpreted. The project proposes an approach for maintaining digital data for hundreds of years through development of an environment that supports migration of collections onto new software systems. The proposed infrastructure combines elements from supercomputer centers, digital libraries, and distributed computing environments. The project emphasizes the synergy that is achieved through the identification of the unique capabilities provided by each environment, and the construction of interoperability mechanisms for integrating these environments. According to this project, collection-based persistent archives are now feasible and can manage massive amounts of information based on XML [49].

### 3.8 InterPARES

The International Research on Permanent Authentic Records Electronic Systems (InterPARES)^12^ project [38] is a multinational research initiative, in which archival scholars, computer engineering scholars, national archival institutions, and private industry representatives are collaborating to develop the theoretical knowledge and methodology required for the permanent preservation of authentic records created using electronic systems [50].

The research areas include identifying the electronic record elements that need to be maintained, developing criteria to appraise electronic records for preservation, and formulating principles for development of international, national, and organizational preservation strategies. Specifically, the research areas are divided into four complementary domains: authenticity, appraisal, preservation, and strategies. In terms of authenticity, the purpose is to identify the specific elements of electronic records that must be preserved, over time and across technologies, in order to verify the record’s authenticity. As a first step, a template to guide the analysis of electronic records was developed and is being evaluated. The project is based in the School of Library, Archival and Information Studies at the University of British Columbia in Canada. The current phase of the project would end on December 31, 2001.

### 3.9 PRISM

The Preservation, Reliability, Interoperability, Security, Metadata (PRISM)^13^ project [29] of the Cornell University is a 4 year project funded by the Digital Library Initiative to investigate and develop policies and mechanisms needed for information integrity in digital libraries. The project focuses on long-term survivability of digital information, reliability of information resources and services, interoperability, security, and metadata.

The current direction of the project is toward developing techniques for monitoring the integrity of distributed Web-based information resources and enforcing preservation policies set by the owners and users of collections. Monitoring resources will involve both the automated capture of information using a specialized Web crawler and the manual gathering of data on the organizational status of particular resources and collections. The ultimate objective is to develop a cost-effective and event-based metadata scheme that will enable users to define preservation policies and enforce them automatically.

### 3.10 Canadian Projects

E-preservation^14^ was developed through a cooperative effort between the National Library of Canada and the Canadian Initiative on Digital Libraries (CIDL). E-preservation is intended 1) to provide Canadians with easy access to policies and 2) to perform research on the creation, use, and preservation of digital collections. The project includes guidelines about various aspects including acquiring digital materials, formats, and metadata.

### 3.11 Preservation Projects at the National Institute of Standards and Technology (NIST)

The earliest work on data preservation at NIST can be traced back to the 1980s when Podio performed research on the lifetime measurement of compact discs [51]. His work provided a basis for a standard methodology for the lifetime measurement of optical discs. With the increasing usage of digital storage in libraries and the archiving of the government agencies, the great importance of digital preservation became clear to NIST’s Information Technology Laboratory,^15^ and accordingly new projects on the study of digital data preservation have started in the following aspects:

- Longevity testing. This project initially consisted of an examination of the effects of heat and humidity on the lifetime of optical discs and was later extended to include the effects of light exposure. The focus is not only on the lifetime itself, but also on the deterioration process. The results may be useful both for new disc production and for the classification of existing recorded discs.
- Testing of interchangeability and interoperability of optical discs for use in high-density storage systems such as optical disc “Jukeboxes.” Combined with the application and further development of XML, a new preservation strategy may be developed. This program is being conducted in collaboration with the High Density Storage Association (HDSA).^16^ An open testing laboratory is being developed that will include interoperability and interchangeability testing as well as testing of the suitability of various types of high capacity storage systems for different applications including preservation.
- Development of the Turbo coding system [52]. Unlike the traditional digital preservation techniques that aim to keep the information readable in the long term, this new technique aims to develop a method for finding and recovering useful information from failed discs. The failure of many commercial error correction codes to support retrieval of important information from aging or damaged digital storage devices has indirectly resulted in the reduction of the rated life expectancy and archival properties of digital storage devices. Turbo code has shown promise in retrieving information previously inaccessible. Turbo codes are two parallel, recursive, and systematic convolution codes. These codes are used for the channel coding and decoding in order to detect and correct the errors that may occur in the transmission of digital data through different channels. The iterative method of the decoding scheme helps to achieve the theoretical limit (near Shannon-limit [53]) in error correction performance. This program is being conducted in cooperation with Carnegie-Mellon University.

## 4. Discussion and Summary

In this paper, three main preservation techniques have been discussed in detail. Each of them has advantages and disadvantages as shown in Table 1. Different strategies are viable for different types of digital materials. In cases of complex resources and application softwares such as games and executable files, emulation is a suitable approach. In particular, emulation is the most feasible choice where there is a lack of sufficient knowledge regarding the format of the digital information and where the look and feel of digital information is important.

**Fig. 4:**
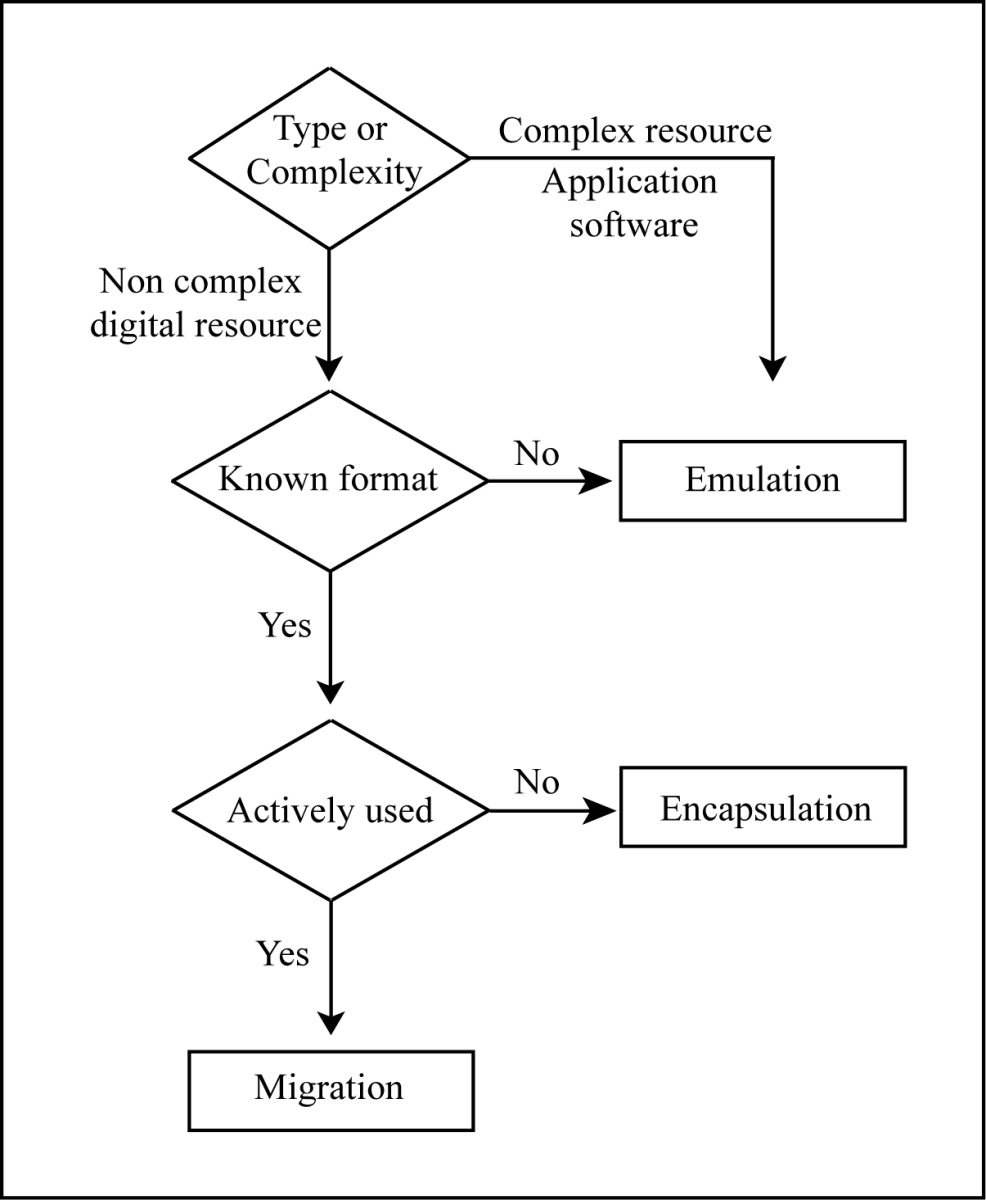
A schematic diagram for selection of preservation techniques of digital information.

On the other hand, migration or encapsulation is appropriate for digital resources where knowledge of the format is sufficient and where the resource is relatively simple. Specifically, migration is appropriate for resources that are actively accessed and managed. Encapsulation is suitable for resources that are unlikely to be actively accessed. Fig. 4 shows a schematic diagram to select the suitable preservation techniques for digital resources according to the type and complexity of digital information, the availability of the data format, and its usage.

As mentioned before, the objective of long-term digital preservation is to ensure continuing access to stored digital information. Future users will be able to access digital information, which will be preserved by digital libraries, through their own computing environments or portable reading devices. This means that the digital information may have to be migrated for long-term digital preservation even if some emulation techniques have already been applied. Therefore, long-term preservation may involve several preservation techniques. In other words, for successful preservation, combined application of different strategies should be taken into account. These techniques are not mutually exclusive, that is, one approach may include some aspects of other approaches.

The preservation of physical artifacts generally occurs through developing and maintaining collections in a decentralized manner. By contrast, the preservation of digital resources does not lend itself well to such a process and no single institution can preserve all digital information. Fortunately, the Internet enables digital libraries worldwide to communicate and share information online. For this to happen successfully, the interoperability of archives and digital library systems and the interchangeability of preserved digital information are necessary.

Standard formats for preservation of digital information should be developed. Standards can provide optimum interoperability and interchangeability, well-constructed tool sets for developers, and solutions for complex systems. Furthermore, standard solutions are likely to be available for use for far longer than nonstandard solutions. Migration from standard formats is much easier, cheaper, and more accurate that migration from non-standard formats.

Standards are required for both emulation and encapsulation. Conventional standards were based on various kinds of encoding schemes according to the types of digital resources, with the result that quite a few software packages support these standards. Meanwhile, XML is being adopted as a standard in various fields due to the recognition of its advantages, including its independence of hardware and software, and due to its wide dissemination. Although XML is a relatively new technology for the Web, SGML (Standard Generalized Markup Language) [54], which is a superset of XML, is a proven technology that has been tested and evaluated since 1986, when it was accepted as an international standard. Furthermore, standards based on XML will facilitate support for the Internet and Web. For example, a standard [55] for digital representation of paper-based books is based on XML.

Since the use of XML is expected to grow wider in the areas of interoperability and interchangeability, preservation standards based on XML are needed. A single standard format is desirable when implementing a preservation system.

The most successful preservation strategies will contain elements of migration based on standardization. While techniques for the migration of digital resources comprised of simple data appear to be widely accepted and followed, the preservation community is only beginning to address migration of more complex digital objects. Additional research on migration is needed to test the technical feasibility of various approaches to migration, determine the costs associated with these approaches, and establish benchmarks and best practices.

Furthermore, a test bed is needed in which specific migration techniques can be prototyped. This test bed should be used to quantitatively test the performance of migration tools that are currently available on the market and new tools being developed. Results from this test bed shall give good guidelines for choosing appropriate tools to migrate digital resources that are in different formats. For this purpose, systematic categorization of digital resources must be developed.

Other critical technical issues must be resolved for preserving digital information. They include requirements relating to metadata, authenticity and integrity, cost modeling, content and structure, format and styles, storage media, and workflow process. Brief descriptions of some critical issues are as follows:

### Metadata

The metadata for digital preservation is critical but complex. Metadata types include descriptive metadata for resource discovery, administrative metadata for the preservation process itself, technical metadata, and rights metadata to describe copyright information [3]. Metadata must enable access to the intellectual content of the object (whether by migration or emulation), find the object, manage the object, and allow other versions of the object to be produced. There have been several specifications for preservation metadata by CEDARS [56], the National Archives and Library of Australia [57,58], and NEDLIB [59]. Recently, collaborative efforts between RLG and the Online Computer Library Center (OCLC) have produced a report [60] identifying common goals and approaches to these digital preservation metadata. The metadata should cover the requirements of various types of organizations and be standardized for interoperability. Efforts to develop tools for automatically creating metadata are needed.

### Interoperability and Interchangeability

As mentioned before, the interoperability of archives and digital libraries as well as the interchangeability of digital information are important issues, particularly in a distributed environment such as the Internet. Standard formats and procedures should therefore be developed.

### Authenticity and Integrity

Authentication allows the user to be certain of the originality of the digital resource when it is needed. Although there are several techniques used for authentication including cryptography, hashing, and time stamping [15], there are multiple meanings and implications of authenticity and integrity. Therefore, creating a common understanding of authenticity and integrity is critical in the digital environment [61]. Dollar [62] identifies maintaining the authenticity of a digital object as a key aim in the migration process.

### Cost Model

Decisions about preserving information should consider the costs [6]. However, there are no proven techniques for estimating the costs of long-term preservation of digital information. Recently, Russell and Weinberger [63] postulate that the ongoing costs of digital preservation span a more extended timeframe than traditional preservation and will therefore require resource commitments of a different nature. Different strategies may necessitate different costing timeframes and schedules. They state that current cost models have yet to reflect this more complex environment.

## Figures and Tables

**Table 1:** Advantages and disadvantages of preservation techniques

Technique	Advantages	Disadvantages	Domain
Emulation	Maintains the look and feel.	The complexity of creating emulator specifications. The large amount of information that must be preserved. Archaic software required to access information.	Application software. Complex digital resources such as those that contain executable files. Resources for which there is a lack of sufficient knowledge. Resources for which the value is unknown and for which future use is unlikely. Resources whose look and feel are important.
Migration	Does not need to retain original applications. Supports active access and management.	Significant cost for long-term preservation. Information degradation. Lack of preservation metadata. Need for continued diligence on the part of archivists.	Resources that are actively accessed and managed, such as scientific data or database. Resources whose formats are sufficiently well known.
Encapsulation	Maintains preservation information.	Knowledge about the format must be preserved. Systems required for capturing the digital information.	Resources that are unlikely to be accessed and managed actively. Resources whose formats are sufficiently well-known.

## Footnotes

1 http://www.konbib.nl/nedlib/

2 http://www.ccsds.org/

3 http://www.si.umich.edu/CAMILEON/

4 http://www.leeds.ac.uk/cedars/

5 Certain commercial equipment, instruments, or materials are identified in this paper to foster understanding. Such identification does not imply recommendation or endorsement by the National Institute of Standards and Technology, nor does it imply that the materials or equipment identified are necessarily the best available for the purpose.

6 http://www.prov.vic.gov.au/vers/

7 http://www.nla.gov.au/policy/plan/pandora.html

8 http://kulturarw3.kb.se/html/projectdescription.html

9 http://www.archive.org/

10 http://sunsite.berkeley.edu/

11 http://www.sdsc.edu/NARA/

12 http://www.interPARES.org/

14 http://www.nlc-bnc.ca/cidl/preserv-conserv/index.htm

15 http://www.itl.nist.gov/

16 http://www.hidensity.org/

## 5. Appendix A. Glossary

The following is a list of important words that are used in this paper. Brief definitions of these words are summarized here for readers’ convenience. For more specific definitions refer to the URLs.

### Emulation

The process of setting up a system to perform in the same way as another system of a different type in order to run its programs.

<http://www.nla.gov.au/download/dsp/appendices.pdf>

### Encapsulation

A technique of grouping together a digital object and anything else necessary to provide access to that object.

<http://www.nla.gov.au/padi/topics/20.html>

### Migration

The periodic transfer of digital materials from one hardware/software configuration to another or from one generation of computer technology to a subsequent generation. The purpose of migration is to preserve the integrity of digital objects and to retain the ability for clients to retrieve, display, and otherwise use them in the face of constantly changing technology.

<http://info.wgbh.org/upf/glossary.html>

### Open Archiving Information System (OAIS)

An archive, consisting of an organization of people and systems, that has accepted responsibility to preserve information and make it available for one or more designated communities.

<http://www.kb.nl/coop/nedlib/glossary.pdf>

### Refreshing

To copy digital information from one long-term storage medium to another of the same type, with no change whatsoever in the bit − stream (e.g. from a decaying 800bpi tape to a new 800bpi tape, or from an older 5 1/4” floppy to a new 5 1/4” floppy).

<http://info.wgbh.org/upf/glossary.html>

### XML (eXtensible Markup Language)

Subset of SGML designed to be transmissible over the Internet in such a way that document browsers do not need to access the document type definition to validate the document before display. As well as requiring all elements to be “well-formed,” e.g., have both start and end tags present, the specification provides XML specific attributes and processing instructions that can be used to control the way documents are presented to users.

<http://www.w3.org/TR/PR-xml.html>
